# Extensive Biotransformation Profiling of AZD8205, an Anti-B7-H4 Antibody-Drug Conjugate, Elucidates Pathways Underlying Its Stability *In Vivo*

**DOI:** 10.1021/acs.analchem.4c02309

**Published:** 2024-10-11

**Authors:** Yue Huang, Hui Yin Tan, Jiaqi Yuan, Ruipeng Mu, Junyan Yang, Kathryn Ball, Balakumar Vijayakrishnan, Luke Masterson, Krista Kinneer, Nadia Luheshi, Meina Liang, Anton I. Rosenbaum

**Affiliations:** † Integrated Bioanalysis, Clinical Pharmacology and Safety Sciences, R&D, AstraZeneca, South San Francisco, California 94080, United States; ‡ Clinical Pharmacology and Quantitative Pharmacology, Clinical Pharmacology and Safety Sciences, R&D, AstraZeneca, Cambridge CB21 6GH, United Kingdom; § TTD, Oncology R&D, AstraZeneca, London E1 2AX, United Kingdom; ∥ Translational Medicine, Oncology R&D, AstraZeneca, Gaithersburg, Maryland 20878, United States; ⊥ Oncology R&D, AstraZeneca, Cambridge CB2 8PA, United Kingdom

## Abstract

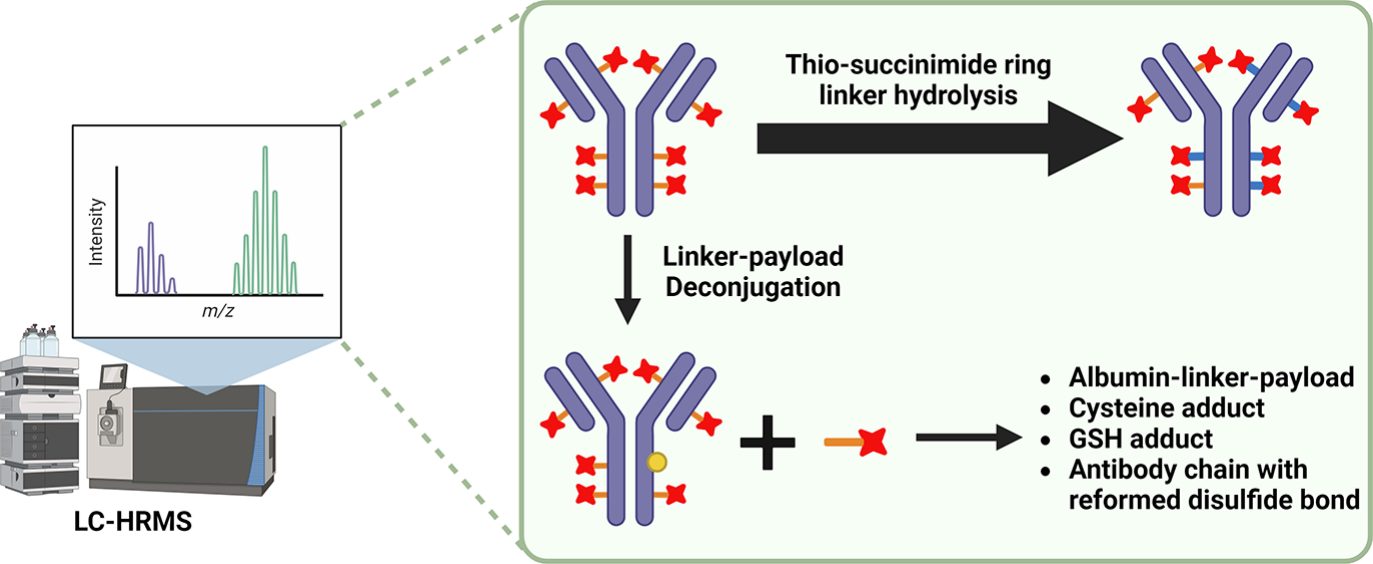

What happens to macromolecules *in vivo*? What drives the structure–activity relationship and *in vivo* stability for antibody-drug conjugates (ADCs)? These interrelated questions are increasingly relevant due to the re-emerging importance of ADCs as an impactful therapeutic modality and the gaps that exist in our understanding of ADC structural determinants that underlie ADC *in vivo* stability. Complex macromolecules, such as ADCs, may undergo changes *in vivo* due to their intricate structure as biotransformations may occur on the linker, the payload, and/or at the modified conjugation site. Furthermore, the dissection of ADC metabolism presents a substantial analytical challenge due to the difficulty in the identification or quantification of minor changes on a large macromolecule. We employed immunocapture-LCMS methods to evaluate *in vivo* changes in the drug-antibody ratio (DAR) profile in four different lead ADCs. This comprehensive characterization revealed that a critical structural determinant contributing to the ADC design was the linker, and competition of the thio-succinimide hydrolysis reaction over retro-Michael deconjugation can result in superb conjugation stability in vivo. These data, in conjunction with additional factors, informed the selection of AZD8205, puxitatug samrotecan, a B7-H4-directed cysteine-conjugated ADC bearing a novel topoisomerase I inhibitor payload, with durable DAR, currently being studied in the clinic for the potential treatment of solid malignancies (NCT05123482). These results highlight the relevance of studying macromolecule biotransformation and elucidating the ADC structure–*in vivo* stability relationship. The comprehensive nature of this work increases our confidence in the understanding of these processes. We hope this analytical approach can inform future development of bioconjugate drug candidates.

## Introduction

Over the past decades, structural characterization of macromolecules *in vitro* has advanced significantly. A plethora of techniques have been employed to characterize the structure of a protein macromolecule primary sequence as well as secondary and tertiary structures at atomic and subatomic resolution. Advanced techniques have been applied to characterize molecular dynamics of molecules^1,2^ and recent advances have focused on the characterization of macromolecular complexes and noncovalent interactions.^3,4^ In the case of small molecules, the structural characterization *in vitro* has been extended to the *in vivo* realm under the auspices of biotransformation analyses. Decades of research into the biotransformation of small-molecule xenobiotics enriched our understanding of such processes. However, the characterization of changes to *protein* macromolecule structure *in vitro* as well *in vivo*, i.e., biotransformation, is an emerging area of scientific inquiry.^5,6^ The main biotransformation pathway for traditional protein therapeutics such as monoclonal antibodies (mAbs) usually involves straightforward proteolysis.^7^ Therefore, recent work in macromolecule biotransformation has focused primarily on the characterization of complex biotherapeutics such as antibody-drug conjugates (ADCs).^8−10^

ADCs combine the high specificity of monoclonal antibodies and potent cytotoxic drugs connected by a cleavable or noncleavable linker for targeted drug payload delivery.^11^ Presently, 15 ADCs have obtained approval from the Food and Drug Administration (FDA) or the European Medicines Agency (EMA).^12,13^ ADCs are typically designed to stay intact while in circulation and release their drug payload upon target-mediated internalization into tumor cells, maximizing the therapeutic index (TI). The linker design plays a major role in modulating the timing and location of drug release.^14^ However, biotransformation of ADCs, such as payload deconjugation or modification to the antibody, drug, or linker can impact their *in vivo* stability.^7,15^ Hence, in-depth characterization of ADC biotransformations would aid in their chemical optimization, influencing *in vivo* stability.

Bioanalytical strategies for the quantification and characterization of novel bioconjugate therapeutics have been thoroughly discussed over the past several years.^6,16^ Typical approaches for ADC quantification in support of pharmacokinetic assessments entail monitoring of surrogate analytes (peptides/payloads) via a targeted bottom-up approach. Therefore, information on biotransformation can be lost without *a priori* knowledge. High-resolution accurate mass spectrometry (HRMS)-based intact analysis of ADCs coupled with chromatographic separation is a powerful and robust tool for the identification of novel biotransformation species. Recent advances in the field of HRMS in addition to more efficient ionization of macromolecules enable the progress of analyzing intact biotherapeutics such as mAbs and ADCs.^17−20^ In addition, other approaches such as CE, HIC, and SEC coupled with MS have been employed as well. Han et al.^21,22^ reported case studies with CE-MS applied to protein biotransformation analysis. He et al.^17^ pioneered ADC biotransformation analyses using the RPLC-MS approach. Additional applications of HIC-MS^23,24^ and SEC-MS^25^ suggest that alternative approaches can be explored for structural analyses of ADCs.

**Figure 1:**
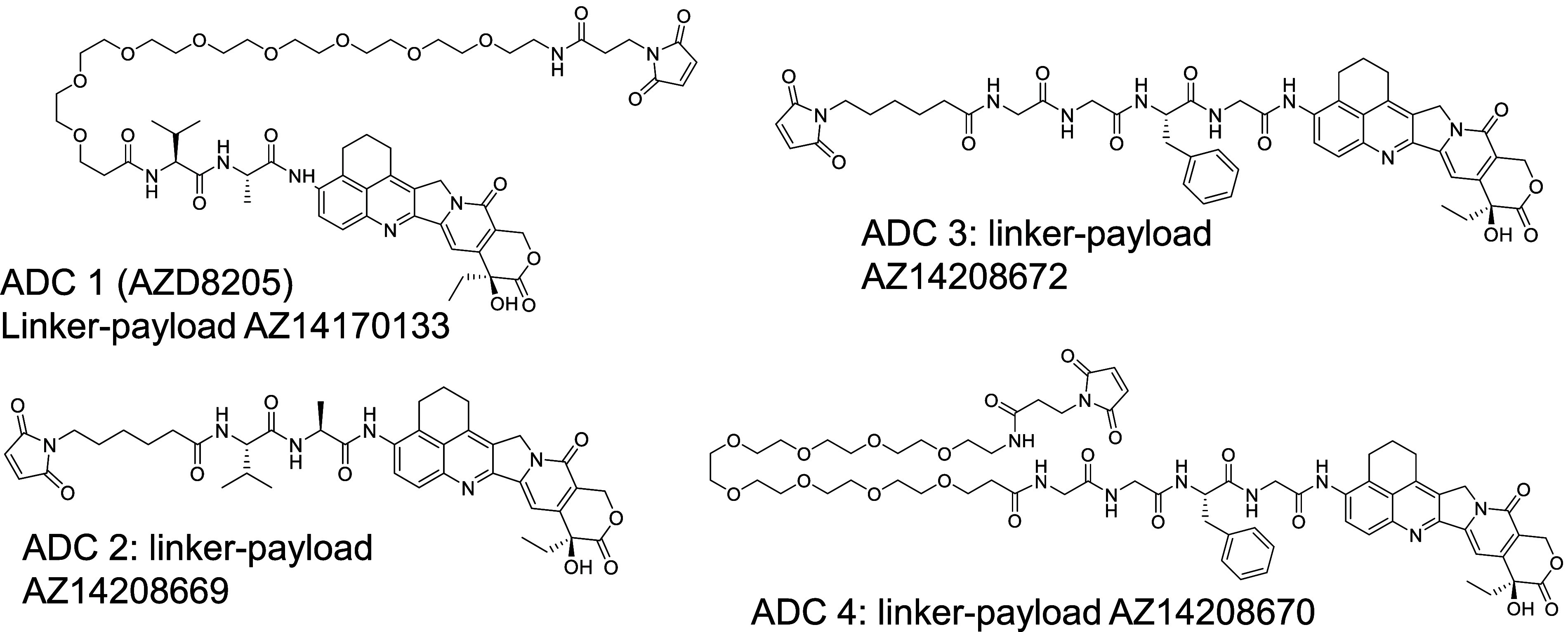
Schematic representation of the linker-payload structures evaluated.

AZD8205, puxitatug samrotecan, is a B7-H4-targeted ADC utilizing a novel topoisomerase I inhibitor linker-payload^26^ (Figure 1) being studied in the clinic for the treatment of biliary tract, breast, ovarian, or endometrial cancers (NCT05123482).^27,28^ As part of the structure–activity relationship (SAR) optimization of AZD8205, we examined four different linkers to enable the conjugation of the topoisomerase I inhibitor payload (TOP1i AZ14170132).^27^ The payload was covalently conjugated to native interchain cysteines of an anti-B7-H4 antibody via either a caproyl or propionyl-PEG8 spacer to a Val-Ala (VA) or Gly-Gly-Phe-Gly (GGFG) peptide linker (Figure 1), resulting in four distinct anti-B7-H4 ADCs, each with an approximate drug-to-antibody ratio (DAR) of 8. To characterize AZD8205 pharmacokinetics and biotransformation using both *in vitro* incubation and *in vivo* plasma samples in mice dosed with AZD8205, we employed intact and bottom-up approaches. Herein, we describe the comprehensive characterization of pharmacokinetics and biotransformation of these ADCs from both *in vitro* and *in vivo* samples, employing orthogonal approaches that provide complementary information. The findings confirmed the durable structural and conjugation stability of AZD8205 among the four linker designs evaluated.

## Experimental Section

### Materials and Reagents

All ADCs, payload, and stable isotope labeled payload, anti-idiotype, and anti-payload antibodies used were provided by AstraZeneca (Gaithersburg, MD). Anti-human-Fc capture antibody was purchased from Bethyl. Peptide internal standards were custom-synthesized by Vivitide. The pooled plasma was purchased from BioIVT. The SMART IA magnetic beads, tris(hydroxymethyl)aminomethane (Tris) buffer, phosphate-buffered saline (PBS), Zeba 7K MWCO spin column, formic acid (FA), trifluoroacetic acid (TFA), and sulfo-NHS biotin were all purchased from Thermo Scientific. Bovine serum albumin and papain were purchased from Sigma-Aldrich. The chromatographic columns (BEH C18 and BioResolve) were purchased from Waters. All other reagents were purchased from VWR.

### LBA-LC-MRM Method for Quantification of Total Antibody, Intact Antibody, and ADC Concentration

Calibration curve standards and quality control samples were prepared in blank pooled CD-1 mouse plasma by using reference standard AZD8205. Calibration range: 0.100–15.0 μg/mL. A 50 μL portion of the sample was then enriched by immunoaffinity capture using 30 μL of SMART IA streptavidin beads conjugated to 3 μg of biotinylated anti-human-Fc antibody upon approximately 2 h incubation at ambient temperature. After the beads were separated from the supernatant and after extensive washing of the beads, SMART IA digestion buffer with stable isotope-labeled internal standard was added to the beads for tryptic digestion at 70 °C for 2 h. After trypsin digestion, one fraction of the supernatant was used for the total antibody and intact antibody assays. The other fraction was further digested using 0.5 mg/mL papain (overnight at 37 °C) to release AZ14170132 for the ADC assay. The characteristic peptides were quantified as surrogate analytes for the total antibody (heavy chain peptide, GLEWIGEINHSGSTSYNPSLK) and intact antibody (light chain peptide, NDVGWYQQKPGK) concentrations, and the released AZ14170132 served as the surrogate analyte for the ADC concentration. Internal standards used were stable isotope labeled peptides (terminal lysine ^13^C_6_, ^15^N_2_) and payload (^2^H_5_). The ADCs were immunocaptured via an antibody against the heavy chain. Total antibody assay monitors heavy chain peptide, whereas intact antibody monitors light chain peptide. The presence of a light chain confirmed that the ADC was intact since there were no interchain disulfide bonds in these DAR8 ADCs. The ADC concentration included all species of biotransformed molecules with payload in a DAR-sensitive manner, regardless of linker biotransformations. All three assays were analyzed on a SCIEX Triple Quadrupole 6500+ mass spectrometer coupled with a Shimadzu liquid chromatography system. Chromatographic separation was performed using a Waters ACQUITY UPLC BEH C18 Column (PN186002350). Mobile phases were A: 0.1% formic acid in water and B: 0.1% formic acid in acetonitrile with a flow rate of 0.5 mL/min at 60 °C. Data were acquired and analyzed with Analyst (v1.7) and MultiQuant (v3.0.3863) software, respectively.

### Intact LBA-LC-HRMS Profiling of Biotransformation Species

**Figure 2:**
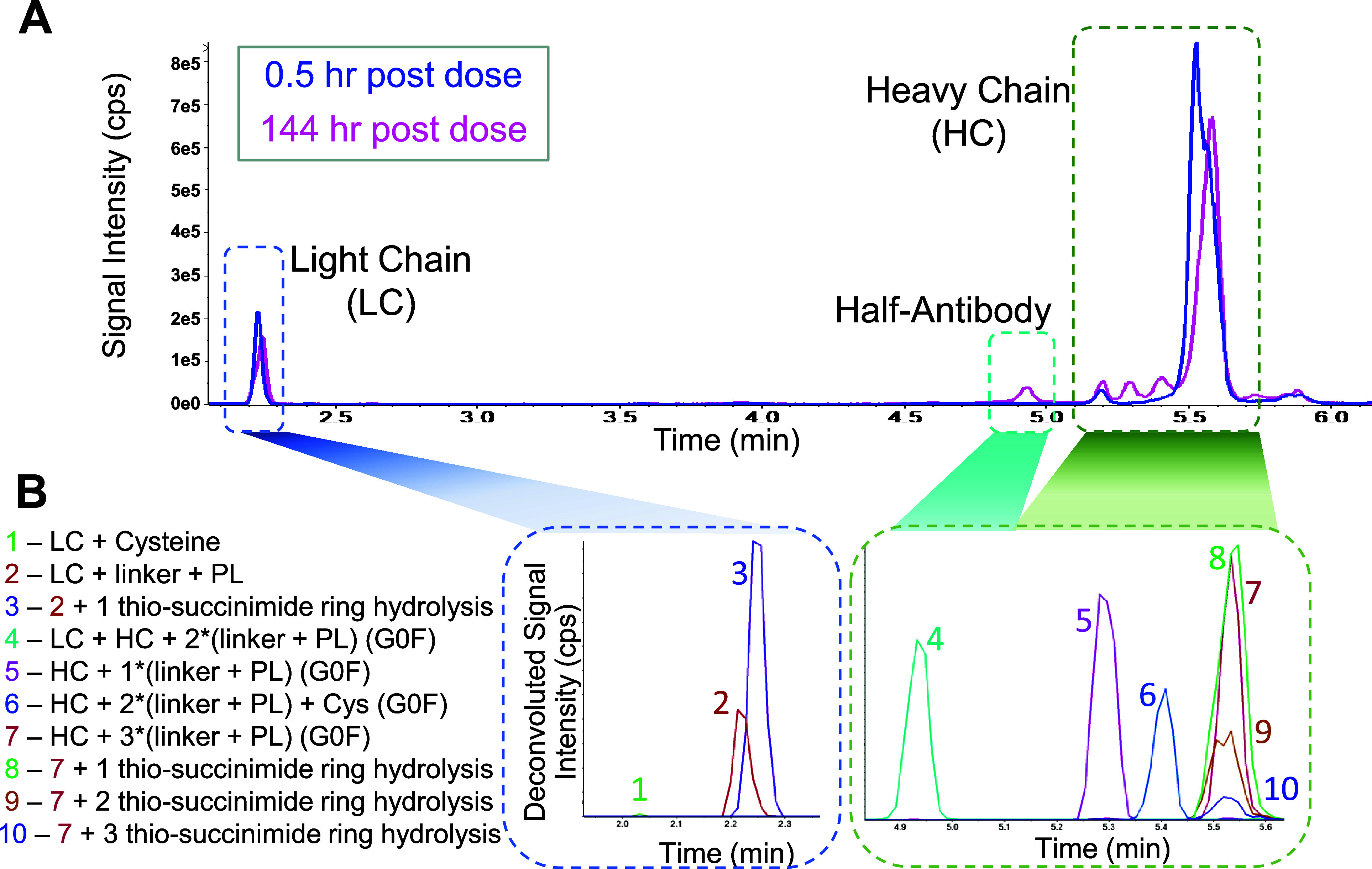
(A) Representative total ion chromatograms for the ADCs, 0.5 and 144 h post dose in humanized FcRn mice. (B) Extracted ion chromatograms for selected major biotransformation species from deconvoluted data (mass–time) of peaks identified in A. LC: light chain, HC: heavy chain, PL: payload, Cys: Cysteine.

An intact LBA-LC-HRMS assay was developed to characterize ADC biotransformation species from *in vitro* and *in vivo* samples. This method allows for a more specific identification of various biotransformation species as well as unbiased quantification. For each sample, the ADC concentration was first measured with the LBA-LC-MRM assay. Plasma concentrations were then adjusted to achieve 8.3 μg/mL ADC with a 120 μL aliquot enabling capture of 1 μg ADC. For certain samples with low concentrations where 1 μg of ADC was not achievable, the maximum volume of original plasma available was used (Table S1) in the capture step. The plasma sample and 75 μL of SMART IA magnetic beads conjugated with 12.5 μg of biotinylated anti-human Fc (a-HuFc) or anti-payload antibody were incubated for approximately 30 min at ambient temperature to capture the ADC and its biotransformed species. After removal of the supernatant following the capture step, the beads were then washed twice with PBS and then twice with water (250 μL each wash step). Finally, the ADC and biotransformed species were eluted off the beads by incubating the beads for 5 min with 45 μL of 1% FA in water with cytochrome C. The samples were not deglycosylated or reduced to preserve the maximal information for the identification of biotransformation species. The eluted samples were injected into Shimadzu Nexera LC. The separation was performed on a Waters BioResolve RP mAb polyphenyl column (PN186009017) with 1% FA, 0.01% TFA in water/ACN as mobile phases with a flow rate of 0.5 mL/min at 80 °C (example chromatogram in Figure 2A). Under the denaturing conditions of reversed-phase liquid chromatography, DAR8 ADC light chain and heavy chain would separate due to the replacement of interchain disulfide bonds with linker-payloads. A shallow gradient was applied during reversed-phase separation to resolve the various species and the major parent molecule. After LC, the separated species were then ionized and acquired in full scan mode with either the SCIEX 6600 Triple TOF or 7600 Zeno TOF system.

### Deconvolution, Identification, and Quantification of Intact LBA-LC-HRMS Data

The mass spectra were deconvoluted using PeakView (research version number: 1.2.2.0) with a sliding window method (Figure S1). This approach converts every three spectra within the *m*/*z* time domain to deconvoluted spectra in the mass–time domain. This preserves chromatographic features, such as retention time. This automated method treated all of the data consistently, eliminating analyst bias in peak selection. This deconvolution method also eliminated the potential impact from the neighboring main peak with high signal intensity on the smaller biotransformed peaks with lesser signal intensity. The mass–time information was then used to manually identify the biotransformed species structures. To quantify the relative abundance of the various biotransformed species, the mass–time chromatograms were analyzed with MultiQuant software using automatic peak integration (MQ4) at the theoretical mass with ±50 ppm as the extraction range. Prespiked cytochrome C was used to monitor run performance. The extracted peak area of each species was normalized with injected ADC mass for comparison between time points. For relative quantification of biotransformation species (% species) at each time point, the percentage was calculated by dividing the sum peak area of a class of species that shared a common feature (e.g., all heavy chain species with G0F) by the sum peak area of all biotransformation species in that class, including parent species (e.g., all heavy chain species).

## Results and Discussion

### Characterization of Pharmacokinetics with LBA-LC-MRM

The most common approach to understanding macromolecule biotransformation is to use a surrogate analyte method and measure fragments from the region of interest to indirectly confirm the structural integrity of the macromolecule.^6,29^ This was performed with LBA-LC-MRM method for all ADCs in this study. This approach was used to generate absolute quantitative results for the total Ab, intact Ab, and ADC assays (Figure S2, Table S2). The data generated using the three methods resulted in overlapping concentration–time profiles for all four ADCs, suggesting that no significant deconjugation was observed, and the protein scaffold remained stable. The differences in concentration–time profiles between the four ADCs with various linkers were not significant when characterized with LBA-LC-MRM assays, considering the 20% accuracy and precision acceptance criteria for the assays.

### Determination of *In Vivo* Biotransformation Pathway Informs AZD8205 Lead Selection

To understand the impact different cleavable linkers would have on *in vivo* DAR stability, we further examined ADC biotransformation pathways using the LBA-LC-HRMS approach. In a DAR8 ADC where the interchain disulfide bonds were replaced by the linker-payload conjugation, the light chain and the heavy chain are not covalently bonded and would separate under denaturing conditions of reversed-phase chromatography. First, as shown in Figure 2A, the light chain and the heavy chain are well separated, with the associated biotransformed species of the light chain and heavy chain eluting close to the parent light chain and the parent heavy chain. Second, to maximize the identification of the various biotransformed products and to facilitate the quantification of these species in an unbiased manner, an automated deconvolution was performed with PeakView (research version), where each spectrum was deconvoluted separately and the *m*/*z*–time raw data were converted to mass–time data. Third, manual identification of the major biotransformed species was performed based on the theoretical intact mass difference between the parent peak and the biotransformed species (Figure 2B, Table S3). Then, extracted peak areas from the chromatograms were used for quantification (Table S3), Lastly, the chemical structures of these proposed biotransformed species (Figure S3) were further confirmed with LBA-LC-MRM^HR^ with collision induced dissociation (CID) using *in vitro* incubation samples that possessed the same biotransformation species (Figures S4–S7).

This approach unveiled various macromolecular biotransformed species from light chain (LC), heavy chain (HC), or half antibody, identified over the 12 day period postdose of each ADC in Tg32 mice (Figure 2, Table 1). To perform relative quantification of complex biotransformation species, the various analytes were clustered based on the relevant characteristics to provide simplified metrics for profiling the *in vivo* mixture of an ADC and its biotransformed species. There are two assumptions for the relative characterization of this data set: (1) capture efficiency and ionization efficiency are reasonably comparable among species used for quantification; (2) data processing is performed uniformly for all species regardless of the signal intensity of the biotransformation species.

**Table 1:** List of Biotransformation Species Identified (LC: Light Chain, HC: Heavy Chain, PL: Payload, Cys: Cysteine, H_2_O: Thio-Succinimide Ring Hydrolysis)

**index**	**monitoring species**	**index**	**monitoring species**	**index**	**monitoring species**
1	LC + 1PL	15	HC + 2PL + G0F + 2H_2_O	29	HC + 3PL + G0F + 2H_2_O
2	LC + 1PL + H_2_O	16	HC + 2PL + G1F + 2H_2_O	30	HC + 3PL + G1F + 2H_2_O
3	LC	17	HC + 2PL + G0F + GSH	31	HC + 3PL + G0F + 3H_2_O
4	LC + Cys	18	HC + 2PL + G1F + GSH	32	HC + 3PL + G1F + 3H_2_O
5	HC + G0F	19	HC + 2PL + G0F + Cys	33	LC + HC + 2PL + G0F
6	HC + G1F	20	HC + 2PL + G1F + Cys	34	LC + HC + 2PL + G1F
7	HC + 1PL + G0F	21	HC + 2PL + G0F + H_2_O + Cys	35	LC + HC + 2PL + G0F + H_2_O
8	HC + 1PL + G1F	22	HC + 2PL + G1F + H_2_O + Cys	36	LC + HC + 2PL + G1F + H_2_O
9	HC + 1PL + G0F + H_2_O	23	HC + 2PL + G0F + 2H_2_Os + Cys	37	LC + HC + 2PL + G0F + 2H_2_O
10	HC + 1PL + G1F + H_2_O	24	HC + 2PL + G1F + 2H_2_O + Cys	38	LC + HC + 2PL + G1F + 2H_2_O
11	HC + 2PL + G0F	25	HC + 3PL + G0F	39	albumin
12	HC + 2PL + G1F	26	HC + 3PL + G1F	40	albumin + Cys
13	HC + 2PL + G0F + H_2_O	27	HC + 3PL + G0F + H_2_O	41	albumin +1PL
14	HC + 2PL + G1F + H_2_O	28	HC + 3PL + G1F + H_2_O	42	albumin +1PL + H_2_O

### Biotransformation Step 1: Hydrolysis or Deconjugation from the Conjugation Site

**Figure 3:**
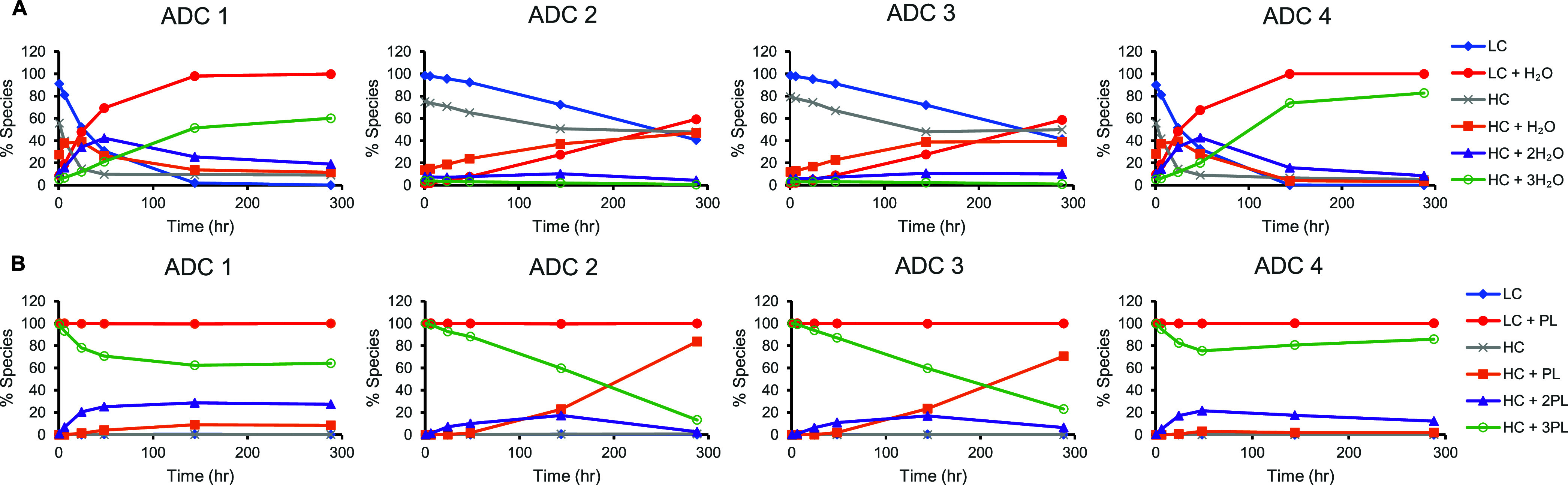
Changes in relative abundance of major biotransformation species for four ADC in mouse preclinical studies as a function of time postdose. (A) Thio-succinimide hydrolysis species. LC includes all LC and LC + 1PL species. HC includes all HC, HC + 1PL, HC + 2PL, and HC + 3PL species. (B) Light and heavy chain species with varied numbers of conjugated payload(s), each species includes all H_2_O states. LC: light chain, HC: heavy chain, PL: payload, H_2_O: thio-succinimide ring hydrolysis. Representative MS1 spectra of these species can be found in Figure S4.

Thio-succinimide-conjugated payloads go through two competing biotransformation reactions: hydrolysis or deconjugation via the retro-Michael reaction.^8^ It was previously demonstrated that some linkers can partially deconjugate, resulting in a protein-partially cleaved payload structure.^9^ Aside from the thio-succinimide ring hydrolysis, protein scaffold instability has also been reported^29^ when the parent molecule has disrupted disulfide bonds. In the case of ADC1 and ADC4, species with a hydrolyzed thio-succinimide ring were the major biotransformation products on both the heavy chain and the light chain (Figure 3A). The retention time of these species did not alter significantly compared to the parent molecule. At 48 h post-dose, the hydrolyzed forms replaced the original species and became the most abundant form of light chain for ADC1 and ADC4 (Figure 3A). For the heavy chain species, the hydrolysis happened gradually: generating partially hydrolyzed species first and then shifting to fully hydrolyzed species. The kinetics of the thio-succinimide hydrolysis is dependent on the chemistry of the linker: both ADC1 and ADC4 contain linkers with propionyl-PEG8 spacers between the amide and the thio-succinimide, resulting in faster hydrolysis rate compared to ADC2 and ADC3 (Figure 3, Figure S8) that contain the caproyl spacer only. The hydrolyzed species were also confirmed with LBA-LC-HRMS bottom-up identification, through both accurate mass MS1 and MS2 spectra (Figure S4 and Table S4). The LC-conjugated linker-payload deconjugates less compared to the HC-conjugated ones for the four ADCs studied here, as observed by other researchers.^12,29^ In contrast to ADC1 and ADC4, for ADC 2 and ADC 3, the deconjugation on HC was observed as the major form, especially for time points after 48 h (Figure 3B). Deconjugation results in species with lower DAR. These species usually elute earlier than the parent molecule. For heavy chain species, the parent heavy chain (with 3 payloads, HC-3PL) eluted around 5.6 min, with the biotransformed species eluting around 5.4 (HC-2PL) and 5.3 min (HC-1PL), respectively (Figure 2B). Note that the degree of deconjugation on LC for all ADCs is consistently low (<0.4%) compared to HC (Figure 3B).

Deconjugation of thio-succinimide-conjugated linker-payloads can result from two possible reactions: (1) retro-Michael elimination at the conjugation site, exposing the free thiol; and (2) stepwise linker cleavage, generating a series of antibody backbone species with partial linker moieties. In the evaluation of ADCs 1–4, only species consistent with reaction 1 deconjugation were observed. Therefore, the deconjugation and thio-succinimide hydrolysis processes for ADCs 1–4 are two competing reactions. There is a clear structure–stability relationship and *in vivo* biotransformation reaction preference observed between the ADCs with propionyl-PEG8 vs caproyl spacer within the linker. This provides a mechanistic basis for improved *in vivo* DAR stability for ADC1 (AZD8205) and ADC4 versus ADC2 and ADC3.

### Relative Quantification of Biotransformed Species

The observed change in ADC DAR postdose enables the evaluation of ADC deconjugation over time. The DAR can be calculated by comparing the total Ab and ADC data from the absolute LBA-LC-MRM quantification (Figure S9A), or with the identified LC and HC species from relative quantification using intact LBA-LC-HRMS (Figures 2 and 3, and Figure S9B). It is notable that both methods, to various extent, showed that ADC1 and 4 had a slower deconjugation rate over time compared to ADC2 and 3. However, the LC-HRMS assay was able to also characterize the structural differences in various species and the kinetics of associated reactions.

### Biotransformation Step 2: Reactions after Deconjugation

**Figure 4:**
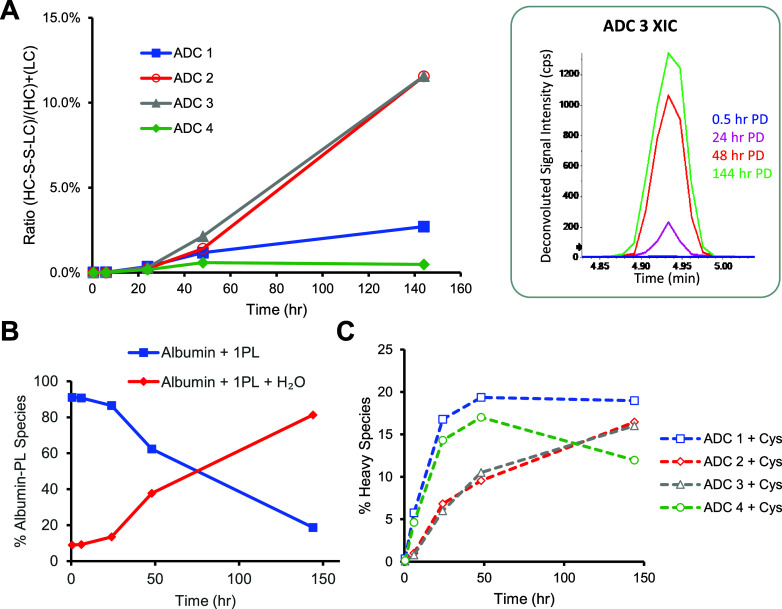
Secondary minor biotransformation reactions after linker-payload deconjugation. (A) Formation of the HC-LC inter chain disulfide bond increases with time; the inset figure showed the extracted ion chromatograph for ADC 3 at various time points. PD = post dose. (B) Cysteinylation changes over time. (C) Quantification of the thio-succinimide hydrolysis of albumin-linker payload. Representative MS1 spectra can be found in Figures S5 and S7.

Upon exposure of free thiols on both heavy chain and light chains following deconjugation, resultant secondary reaction products included cysteine and GSH adducts as well as newly reformed disulfide bonds between spatially close free thiols (Figure 4). This observation is supported with intact mass data and further confirmed with the LBA-LC-MRM^HR^ analyses for selected parent ions (Figures S5–S7). The quantification of these secondary, minor biotransformation species is displayed in Figure 4. Furthermore, the deconjugated small molecule linker-payload has been observed to covalently conjugate to circulating albumin (Table 1, index 41). The albumin-linker-payload can then also undergo thio-succinimide ring hydrolysis (Table 1, index 42).

It is notable that the HC biotransformation species showed a distinct pattern when comparing the loss of one linker-payload to the loss of two linker-payloads. After deconjugation of a single linker-payload, we observed a cluster of peaks with several mass changes (Figure S10C, Table 1, indices 11–24), which are indicative of multiple species formed after the exposed free thiol subsequently reacted with other redox-active molecules in plasma. The observed intact mass matched with the proposed adducts, and the structure was further confirmed with CID MS^2^ spectra (Figure S5). On the contrary, after deconjugation of two linker-payloads, the major observed biotransformed species (Figure S10B, Table 1, indices 7–10) had a mass change consistent with the loss of two linker-payloads (e.g., 2296 Da for AZD8205). No additional secondary adducts were observed. The potential reason for the distinct pattern after two payload losses is the reformation of the intrachain disulfide bond. In the case of the HC-1PL species containing two free exposed thiols and if these thiols are spatially close, reformation of the intrachain disulfide bond becomes the major step 2 reaction. The heavy chain intrachain disulfide bond was confirmed with bottom-up LC-MRM^HR^ (Figure S6).

Alternatively, the reformation of disulfide bonds can happen between the light chain and the heavy chain, following payload deconjugation on both chains. This can be confirmed with the increasing amount of a 76 kDa biotransformation species observed at a retention time of 4.9 min (Figure 4A, Figure S10A). The observed mass matched with the expected mass of the light chain and heavy chain conjugated complex, with two linker-payloads remaining on the heavy chain and potential thio-succinimide ring hydrolysis. This interchain disulfide bond between the light chain and heavy chain was confirmed with bottom-up LC-MRM^HR^ (Figure S7). For ADCs that deconjugated to a greater extent (ADC2 and ADC3), the potential of reformation of the interchain disulfide bond *in vivo* may have contributed to the stabilization of the protein scaffold (Figures 3B and 4A, Table S2). Interchain disulfide bond reformation was observed for all four ADCs, although it was higher for ADC2 and ADC3 (Figure 4A). This is likely due to the larger degree of deconjugation, catalyzing the reformation of the disulfide bonds. After deconjugation, the deconjugated linker payload can reconjugate to various thiol-containing endogenous proteins.^12^ Capture with anti-payload antibody enables detection and characterization of additional proteins that would contain the reconjugated linker payload, such as albumin. Interestingly, the albumin-conjugated conjugated linker payload continued to hydrolyze over time (Figure 4B). These data are consistent with the hypothesis that the linker hydrolysis needs sufficient time for the reaction to proceed, and the slower elimination half-life of albumin conjugated linker-payload enables this reaction to be observed on nonantibody containing macromolecular species. Further relative quantification analyses were also performed on the data set. The analysis suggested that glycoforms on the heavy chain do not seem to have a significant impact on the biotransformation at the conjugation site (Figure S11). Cysteinylation is the major secondary reaction for the exposed free thiol after deconjugation and also gradually increases over time (Figure 4C).

### Biotransformation Pathways of Thio-succinimide Conjugated ADCs

**Figure 5:**
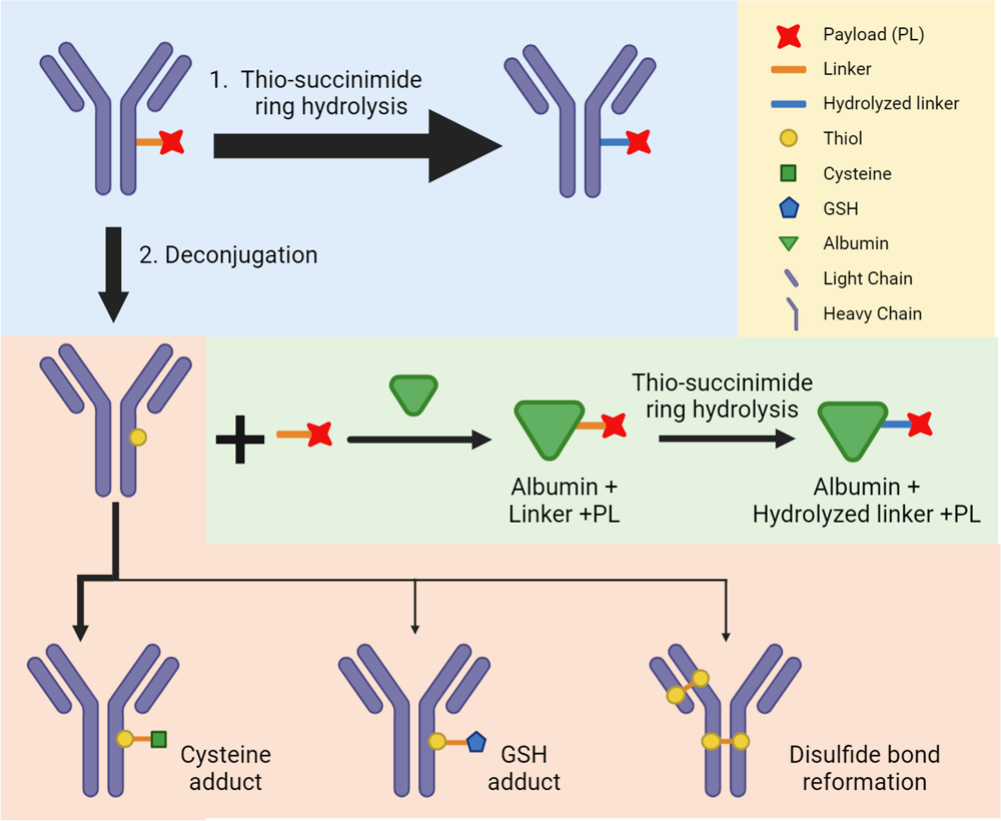
Biotransformation pathway diagram for the 4 ADC studied here. The blue box highlights the first step of biotransformation pathway, where the linker thio-succinimide ring is either hydrolyzed, stabilizing the conjugation, or deconjugated, exposing the free thiol. Following deconjugation, the various components of the ADC can then each go through additional biotransformation reactions. Arrow thickness indicates the preponderance of biotransformation pathways. While all ADCs had an average DAR of ∼8, only one linker-payload per ADC is depicted here for simplicity. Created with BioRender.com.

Consolidating the information comprised of the observed biotransformation species proposed structures, their concentration–time profiles, and common chemical reactions that can be expected under such circumstances, we propose the biotransformation pathways for ADC1–4 depicted in Figure 5. After dosing, the ADC can undergo two competing reactions: (1) hydrolysis on the thio-succinimide linker, further stabilizing the conjugated payload, and (2) deconjugation of the linker-payload, exposing the free thiol. For AZD8205, the vast majority of ADC went through reaction 1 as the main biotransformation pathway, ensuring its *in vivo* stability. Upon deconjugation, further minor biotransformation products were identified. This represents a very small, albeit analytically interesting fraction of the circulating ADC pool.

Understanding the underlying mechanisms behind the ADC biotransformation is critical to advance the drug candidate through discovery and development.^30^ While for small molecule drug candidates ADME studies and metabolite ID analyses are routinely performed, the biotransformation analysis of therapeutic proteins is technically highly challenging. Nonetheless, there is an urgent need to understand the comprehensive biotransformation profile of therapeutic proteins because of the rapidly increasing diversity of complex therapeutic protein formats and the resulting knowledge gap in connecting SAR to drug efficacy and safety.

Biotransformation assessments for therapeutic proteins to date have largely focused on characterizing amino acid post-translational modifications (PTMs) located in critical regions, proteolytic degradation, and glycation or glycosylation^30^. For ADCs, linker-payload stability is often the focus of biotransformation characterization.^14,17,18^ Payload chemical modifications such as deacetylation, adduct formation, and partial cleavage have been reported. However, the existing gaps in our understanding of ADC SAR make it essential to further elucidate the ADC biotransformation profile of the three critical components determining ADC SAR: the protein scaffold, the conjugation site, and the conjugated payload.

For ADCs, SAR is not only dependent on the binding properties of the CDRs, but also heavily related to the characteristics of the conjugation site and chemistry, linker, and payload.^12,14,15,26^ The comprehensive profiling of AZD8205 biotransformations demonstrated that the critical structural determinant contributing to the design of this ADC was the linker structure that either contained propionyl-PEG8 or caproyl spacers. The linker containing the propionyl-PEG8 spacer resulted in an increased thio-succinimide hydrolysis rate (Figure 3A). While this resulted also in an initial increase in deconjugation rate, after approximately 72 h postdose the deconjugation rate was reduced by the competing thio-succinimide hydrolysis reaction allowing ADC1 (AZD8205) and ADC4 to effectively maintain a high DAR ratio throughout the entire duration of exposure (Figure 3B and Figure S9B). Thus, quantitative biotransformation profiling across time can be an informative tool to assess the impact of various structural components on the ADC *in vivo* stability.

Furthermore, LC-HRMS proved to be able to discriminate more readily between subtle changes in DAR compared to the LC-MRM approach, as shown in Figure S9 with detailed DAR calculations defined by eqs S1–S5. We hypothesize that the *direct* DAR analysis using HRMS is more sensitive than the MRM approach, which relies on enzymatically released payload as a surrogate analyte and measures average DAR indirectly. Importantly, both approaches showed that AZD8205 remained very stable in vivo.

Building upon our comprehensive characterization of AZD8205 biotransformation, we can glean the various biochemical reactions that enable us to obtain a more mechanistic understanding of ADC biotransformation pathways. This is particularly important from a translational ADME point of view, as such knowledge would be important to understand the translatability of PK and PD data between animal models and the clinical setting. In addition to the known role of proteases in protein degradation, other endogenous molecules and microenvironments may influence ADC biotransformation. One avenue for interrogating the mechanisms of ADC biotransformation is by examining the endogenous molecules covalently and noncovalently associated with ADCs or their catabolites. Redox pairs, such as cysteine and GSH have been observed to interact with AZD8205 and its catabolites. Further understanding of the determinants behind these interactions may provide further supporting evidence in translating these preclinical study results to patients.

Understanding drug metabolism is a critical component for successful drug development.^5^ Complex macromolecules such as ADCs present unique challenges to gain such an understanding. Therefore, we developed and employed several analytical approaches to profile ADC biotransformation in circulation comprehensively. The results help to better understand factors affecting the underlying pharmacokinetic profiles of the various molecular species which are formed when AZD8205 is administered *in vivo*, and thereby aid in developing a better understanding of SAR for thio-succinimide-linked ADCs. In the future, these findings could better inform the translation of PK/PD from animal models to the clinical setting. Biotransformation profiling of protein conjugates can be further studied in patient populations or in specific organs/tissues with this or a similar method.

## Conclusions

We have presented a comprehensive profiling approach focused on the *in vivo* biotransformation pathway for a series of cysteine-conjugated ADCs with differing linkers. We employed immuno-affinity capture enrichment, coupled with LC-HRMS and complemented with LC-MRM^HR^ confirmation, to obtain characterization data at the protein subunit level. The HRMS data were interpreted with an unbiased deconvolution method. Key biotransformation species were identified with intact mass and bottom-up approaches, and relative quantification was performed based on the peak area. The elimination of the parent molecule and generation of the biotransformed species as a function of time postdose was used to map the biotransformation reaction pathway of the ADC molecules. When applying this methodology in concert to a group of ADCs, the structure–stability relationship was established substantiating the importance of the linker structure for this ADC conjugation approach. These data, along with additional information, reported elsewhere^26^ led to the selection of AZ14170133 as the optimal linker payload resulting in AZD8205 ADC, puxitatug samrotecan. To expand our future understanding of bioconjugate and catabolite interactions with endogenous molecules, we will need to apply this methodology, across bioconjugates with varying conjugation approaches, linkers, and payloads. Notably, it will also be important to evaluate biotransformation pathways in various microenvironments in specific organs, tissues, and tumors to fully understand the determinants for their efficacy and safety profiles. Eventually, the biotransformation information obtained in animal models or *in vitro* experiments may be translated to potential patient populations as part of clinical studies.

## Supporting Information Available

Additional experimental details, pharmacokinetic data, and parameters, further details on biotransformation species and their structures, comparison of relative quantification for DAR using LBA-LC-MRM and LBA-LC-HRMS methods, MS/MS spectra, and quantification of biotransformation species (PDF)
