# Supplementary material for: Extensive Biotransformation Profiling of AZD8205, an Anti-B7-H4 Antibody-Drug Conjugate, Elucidates Pathways Underlying Its Stability In Vivo
Source: Anal Chem. 2024 Oct 11;96(42):16525–33. doi: 10.1021/acs.analchem.4c02309 (PMC11503519; doi:10.1021/acs.analchem.4c02309)
Supplement: Supplementary file 1 — ac4c02309_si_001.pdf [file ac4c02309_si_001.pdf]

Supporting Information

Extensive Biotransformation Profiling of AZD8205, an Anti-B7-H4 Antibody-Drug Conjugate, Elucidates Pathways Underlying its Stability In Vivo

Yue Huang[1*], Hui Yin Tan[1], Jiaqi Yuan[1], Ruipeng Mu[1], Junyan Yang[1], Kathryn Ball[2], Balakumar Vijayakrishnan[3], Luke Masterson[3], Krista Kinneer[4], Nadia Luheshi[5], Meina Liang[1], Anton I. Rosenbaum[1*]

[1]*Integrated Bioanalysis, Clinical Pharmacology and Safety Sciences, R&D, AstraZeneca, South San Francisco, CA 94080, USA*

[2]*Clinical Pharmacology and Quantitative Pharmacology, Clinical Pharmacology and Safety Sciences, R&D, AstraZeneca, Cambridge CB21 6GH United Kingdom*

[3]*TTD, Oncology R&D, AstraZeneca, London E1 2AX, United Kingdom*

[4]*Translational Medicine, Oncology, AstraZeneca, Gaithersburg, MD 20878, USA*

[5] *Oncology R&D, Cambridge CB2 8PA, United Kingdom*

***Corresponding Authors**

Yue Huang - *121 Oyster Point Blvd, South San Francisco, CA 94080, USA*; Tel: +1-650-481-6801 ;E-mail: yhuang@revmed.com

Anton I. Rosenbaum - *121 Oyster Point Blvd, South San Francisco, CA 94080, USA*; Tel: +1-650-379-3099; E-mail: anton.rosenbaum@astrazeneca.com; anton.rosenbaum.phd@gmail.com

Table of Contents:

Experimental Methods **S3**

Table S1. Mouse plasma sample information for intact LC-HRMS biotransformation experiment design. **S4**

Table S2. Pharmacokinetic parameters of ADC1-4 in Tg32 mice. **S5**

Table S3. Theoretical mass of monitored parent and biotransformation species for deconvoluted mass-time chromatogram generated from PeakView (research version). **S6**

Table S4. Confirmed key fragments from enzymatic digestions of selected biotransformation species using the bottom up LBA-LC-MRM^{HR} method. **S8**

Figure S1. Deconvolution parameters in research version PeakView. **S9**

Figure S2. Concentration-time profiles of ADC1-4 after a single bolus 5 mg/kg IV dose in Tg32 mice analyzed by LBA-LC-MRM. **S10**

Figure S3. Conjugation sites of linker-PL on anti-B7-H4 antibody and structures of parent and biotransformed species. **S11**

Figure S4. Selected thio-succinimide hydrolysis species confirmed with bottom-up LBA-LC-MRM^{HR}. **S12**

Figure S5. Cysteine adducts confirmed with bottom-up LBA-LC- MRM^{HR}. **S13**

Figure S6. Reformation of intra-chain disulfide bonds confirmed with bottom-up LBA-LC-MRM^{HR}. **S14**

Figure S7. Reformation of inter-chain disulfide bonds confirmed with bottom up LBA-LC-MRM^{HR}. **S15**

Figure S8. Thio-succinimide hydrolysis at two time points post-dose for the light chain and heavy chain for ADCs 1-4. **S16**

Figure S9. Relative quantification for DAR with LC-MRM method and intact LC-HRMS method for ADC1-4. **S17**

Figure S10. Deconvoluted mass spectra, zoomed in for LC-HC reformation, heavy chain species with only 1 PL and heavy chain species with 2 remaining PLs. **S19**

Figure S11. Glycosylation profile of ADCs 1-4. **S20**

Experimental Methods

Pharmacokinetic Study in Tg32 Mice

All animal experiments were conducted in a facility accredited by the Association for Assessment and Accreditation of Laboratory Animal Care under the guidelines of AstraZeneca's Institutional Animal Care and Use Committee and appropriate animal research approvals. A single dose of the four ADC drug candidates (**Figure** 1) was administered intravenously to male SCID FcRn-/- hFcRn (32) Tg ("Tg32") mice (Strain #:018441, Jackson Laboratories, Bar Harbor, ME) at 5 mg/kg (n=9 per group). K_2EDTA plasma samples were collected post-dose 0.5, 6, 24, 48, 144, 288 h (n=3 for each timepoint).

Non-compartmental Analysis of ADC Pharmacokinetics

For each ADC, the naïve pooled plasma concentration-time data obtained after a single 5 mg/kg IV administration in Tg32 mice using a sparse sampling design were used to estimate mean pharmacokinetic parameters for ADC, total antibody and intact antibody using noncompartmental analysis in Phoenix WinNonlin version 6.4 (Certara, NJ, USA). Concentrations that were below the limit of quantification of the assay were excluded from the pharmacokinetic parameter analysis. The area under the plasma concentration–time curve (AUClast) was calculated by using the linear-up log-down trapezoidal method, and the maximum serum concentration (Cmax), plasma clearance (CL), volume of distribution at steady state (Vss), and terminal elimination half-life (T1/2, z) were also estimated.

Structural Elucidation of Biotransformation Species with Bottom-Up LC- MRM^{HR}

In vitro incubation of AZD8205 (0.1 mg/mL) in pooled human or CD1 mouse plasma at 37 °C were performed for 0, 6, 24, 72, and 168 h. The samples were then processed similarly to total antibody LC-MRM assay as described in the manuscript. The samples were analyzed on the SCIEX 7600 ZenoTOF mass spectrometer coupled with a Shimadzu liquid chromatography system. Waters ACQUITY UPLC BEH C18 Column (PN186002350) was used for chromatography separation using mobile phases (A: 0.1 % FA in water; B: 0.1 % FA in acetonitrile). Data was acquired in positive IDA mode as well as TOF full scan MS2 mode with CID fragmentation for specific m/z of interest. Further data analysis including structural elucidation was performed with SCIEX OS software (v3.1.5.3945).

Index No.	ADC	dose (mg/kg)	time point (h)	ADC Conc (µg/mL)	Volume for 1 µg (µL)	Pooled Plasma Volume (µL)	Actual Volume (µL)	Actual Amount (µg)
1	ADC1	5	0.5	100.04	10.0	110.0		1
2		5	6	55.68	18.0	102.0		1
3		5	24	32.22	31.0	89.0		1
4		5	48	19.87	50.3	69.7		1
5		5	144	8.31	120.4	NA	120.4	1
6		5	288	3.29	303.6	NA	200	0.66
7	ADC2	5	0.5	115.39	8.7	111.3		1
8		5	6	45.44	22.0	98.0		1
9		5	24	25.46	39.3	80.7		1
10		5	48	20.28	49.3	70.7		1
11		5	144	8.65	115.6	4.4		1
12		5	288	3.51	285.2	NA	200	0.70
13	ADC4	5	0.5	112.63	8.9	111.1		1
14		5	6	54.07	18.5	101.5		1
15		5	24	21.14	47.3	72.7		1
16		5	48	18.64	53.7	66.3		1
17		5	144	2.34	428.2	NA	130	0.30
18		5	288	1.81	552.9	NA	150	0.27
19	ADC3	5	0.5	107.48	9.3	110.7		1
20		5	6	45.55	22.0	98.0		1
21		5	24	24.03	41.6	78.4		1
22		5	48	19.06	52.5	67.5		1
23		5	144	8.70	114.9	5.1		1
24		5	288	2.35	425.2	NA	200	0.47

Table S1. Mouse plasma sample information for intact LC-HRMS biotransformation experiment design. The known plasma concentration from the LC-MRM assay was used to calculate the volume needed for 1 ug of ADC extraction.

ADC	Detection	AUC_{last} day*ug/mL	C_{max} ug/mL	CL mL/day/kg	$T_{1/2,\,z}$ day	V_{ss} mL/kg
ADC1 B7H4-AZ14170133	Conj ADC	136	93.0	34.1	3.48	143
	HC	141	94.7	32.9	3.48	139
	LC	130	98.8	36.5	2.95	135
ADC2 B7H4-AZ14208669	Conj ADC	141	105	31.4	3.77	153
	HC	144	95.8	30.1	4.11	163
	LC	144	98.7	30.9	3.68	148
ADC3 B7H4-AZ14208672	Conj ADC	132	102	35.9	2.97	131
	HC	137	102	34.3	3.13	134
	LC	135	92.8	34.5	3.14	146
ADC4 B7H4-AZ14208670	Conj ADC	123	104	39.1	2.56	117
	HC	128	107	37.5	2.61	116
	LC	134	105	35.7	2.66	115

Table S2. Pharmacokinetic parameters of ADC1-4 in Tg32 mice. AUC: area under the plasma concentration–time curve. Cmax: maximum serum concentration. CL: plasma clearance. Vss: volume of distribution at steady state (Vss). T1/2, z: terminal elimination half-life. Conj: Conjugated

Monitoring Species	Antibody Backbone	PL	Glycosylation	Water	Other Modification	Detection Method		Quantification	Theoretical Mass			
						Intact	Bottom-up		ADC 1	ADC 2	ADC 3	ADC 4
LC + 1PL	LC	1				✓		✓	24643.69	24262.27	24410.39	24791.82
LC + 1PL + H_2O	LC	1		1		✓		✓	24661.69	24280.27	24428.39	24809.82
LC	LC	0				✓		✓	23495.42	23495.42	23495.42	23495.42
LC + Cys	LC	0			Cys	✓		✓	23615.42	23615.42	23615.42	23615.42
HC + G0F	HC	0	G0F			✓		✓	50299.29	50299.29	50299.29	50299.29
HC + G1F	HC	0	G1F			✓		✓	50461.29	50461.29	50461.29	50461.29
HC + 1PL + G0F	HC	1	G0F			✓		✓	51447.56	51066.14	51214.26	51595.69
HC + 1PL + G1F	HC	1	G1F			✓		✓	51609.56	51228.14	51376.26	51757.69
HC + 1PL + G0F + H_2O	HC	1	G0F	1		✓		✓	51465.56	51084.14	51232.26	51613.69
HC + 1PL + G1F + H_2O	HC	1	G1F	1		✓		✓	51627.56	51246.14	51394.26	51775.69
HC + 2PL + G0F	HC	2	G0F			✓		✓	52595.83	51832.99	52129.23	52892.09
HC + 2PL + G1F	HC	2	G1F			✓		✓	52757.83	51994.99	52291.23	53054.09
HC + 2PL + G0F + H_2O	HC	2	G0F	1		✓		✓	52613.83	51850.99	52147.23	52910.09
HC + 2PL + G1F + H_2O	HC	2	G1F	1		✓		✓	52775.83	52012.99	52309.23	53072.09
HC + 2PL + G0F + $2H_2O$	HC	2	G0F	2		✓		✓	52631.83	51868.99	52165.23	52928.09
HC + 2PL + G1F + $2H_2O$	HC	2	G1F	2		✓		✓	52793.83	52030.99	52327.23	53090.09
HC + 2PL + G0F + GSH	HC	2	G0F		GSH		✓		NA	NA	NA	NA
HC + 2PL + G1F + GSH	HC	2	G1F		GSH		✓		NA	NA	NA	NA
HC + 2PL + G0F + Cys	HC	2	G0F		Cys	✓		✓	52715.83	51952.99	52249.23	53012.09
HC + 2PL + G1F + Cys	HC	2	G1F		Cys	✓		✓	52877.83	52114.99	52411.23	53174.09
HC + 2PL + G0F + H_2O + Cys	HC	2	G0F	1	Cys	✓		✓	52733.83	51970.99	52267.23	53030.09
HC + 2PL + G1F + H_2O + Cys	HC	2	G1F	1	Cys	✓		✓	52895.83	52132.99	52429.23	53192.09
HC + 2PL + G0F + $2H_2O$ + Cys	HC	2	G0F	2	Cys	✓		✓	52751.83	51988.99	52285.23	53048.09
HC + 2PL + G1F + $2H_2O$ + Cys	HC	2	G1F	2	Cys	✓		✓	52913.83	52150.99	52447.23	53210.09
HC + 3PL + G0F	HC	3	G0F			✓		✓	53744.10	52599.84	53044.20	54188.49
HC + 3PL + G1F	HC	3	G1F			✓		✓	53906.10	52761.84	53206.20	54350.49
HC + 3PL + G0F + H_2O	HC	3	G0F	1		✓		✓	53762.10	52617.84	53062.20	54206.49
HC + 3PL + G1F + H_2O	HC	3	G1F	1		✓		✓	53924.10	52779.84	53224.20	54368.49

Monitoring Species	Antibody Backbone	PL	Glycosylation	Water	Other Modification	Detection Method		Quantification	Theoretical Mass			
						Intact	Bottom-up		ADC 1	ADC 2	ADC 3	ADC 4
HC + 3PL + G0F + $2H_2O$	HC	3	G0F	2		✓		✓	53780.10	52635.84	53080.20	54224.49
HC + 3PL + G1F + $2H_2O$	HC	3	G1F	2		✓		✓	53942.10	52797.84	53242.20	54386.49
HC + 3PL + G0F + $3H_2O$	HC	3	G0F	3		✓		✓	53798.10	52653.84	53098.20	54242.49
HC + 3PL + G1F + $3H_2O$	HC	3	G1F	3		✓		✓	53960.10	52815.84	53260.20	54404.49
LC + HC + 2PL + G0F	LC + HC	2	G0F			✓		✓	76091.25	75328.41	75624.65	76387.51
LC + HC + 2PL + G1F	LC + HC	2	G1F			✓		✓	76253.25	75490.41	75786.65	76549.51
LC + HC + 2PL + G0F + H_2O	LC + HC	2	G0F	1		✓		✓	76109.25	75346.41	75642.65	76405.51
LC + HC + 2PL + G1F + H_2O	LC + HC	2	G1F	1		✓		✓	76271.25	75508.41	75804.65	76567.51
LC + HC + 2PL + G0F + $2H_2O$	LC + HC	2	G0F	2		✓		✓	76127.25	75364.41	75660.65	76423.51
LC + HC + 2PL + G1F + $2H_2O$	LC + HC	2	G1F	2		✓		✓	76289.25	75526.41	75822.65	76585.51
Albumin		0				✓			65857.00	NA	NA	NA
Albumin + Cys		0			Cys	✓			65977.00	NA	NA	NA
Albumin + 1PL		1				✓		✓	67005.27	NA	NA	NA
Albumin + 1PL + H_2O		1		1		✓		✓	67023.27	NA	NA	NA

LC: light chain, HC: heavy chain, PL: payload, Cys = cysteine; NA = not analyzed

Table S3. Theoretical mass of monitored parent and biotransformation species for deconvoluted mass-time chromatogram generated from PeakView (research version). Peaks were detected and extracted with ± 50 ppm tolerance using MultiQuant. Quantification marked out the species used to generate the relative quantification results in Figures 3, 4, S9B and S11.

Biotransformation	Key Section of the Biotransformation Species	Theoretical m/z	z
LC hydrolysis	GEC+1hydrolyzed linker + 1PL	491.8834	3
HC hydrolysis (Fig S3)	SCDK+1hydrolyzed linker + 1PL	539.9134	3
HC hydrolysis	THT+2*(hydrolyzed linker + PL))	1013.1065	5
HC Cys (Fig S4)	THT+1*(hydrolyzed linker + PL) + 1Cys	803.7987	5
HC GSH	THT+1*(hydrolyzed linker + PL) + 1GSH	841.0115	5
HC intra SS (Fig S5)	THT intra S-S	682.8558	4
LC+HC inter SS (Fig S6)	GEC+SCDKTHT+2*(hydrolyzed linker + PL)	967.4619	6

Table S4. Confirmed key fragments from enzymatic digestions of selected biotransformation species using the bottom up LBA-LC- MRM^{HR} method.

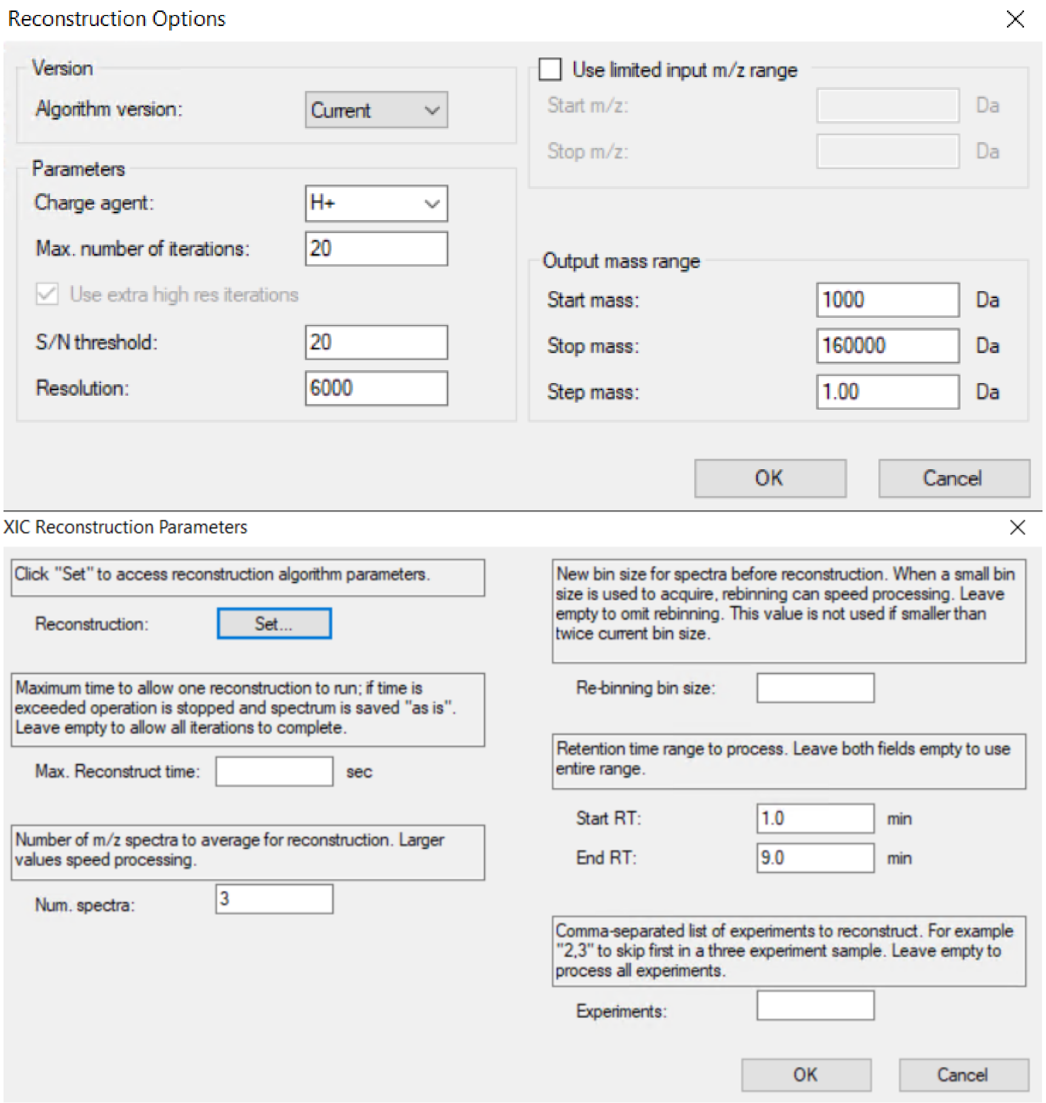

Figure S1. Deconvolution parameters in research version PeakView. The LC-HRMS data is converted from m/z-time to the mass-time domain, while maintaining the chromatographic information.

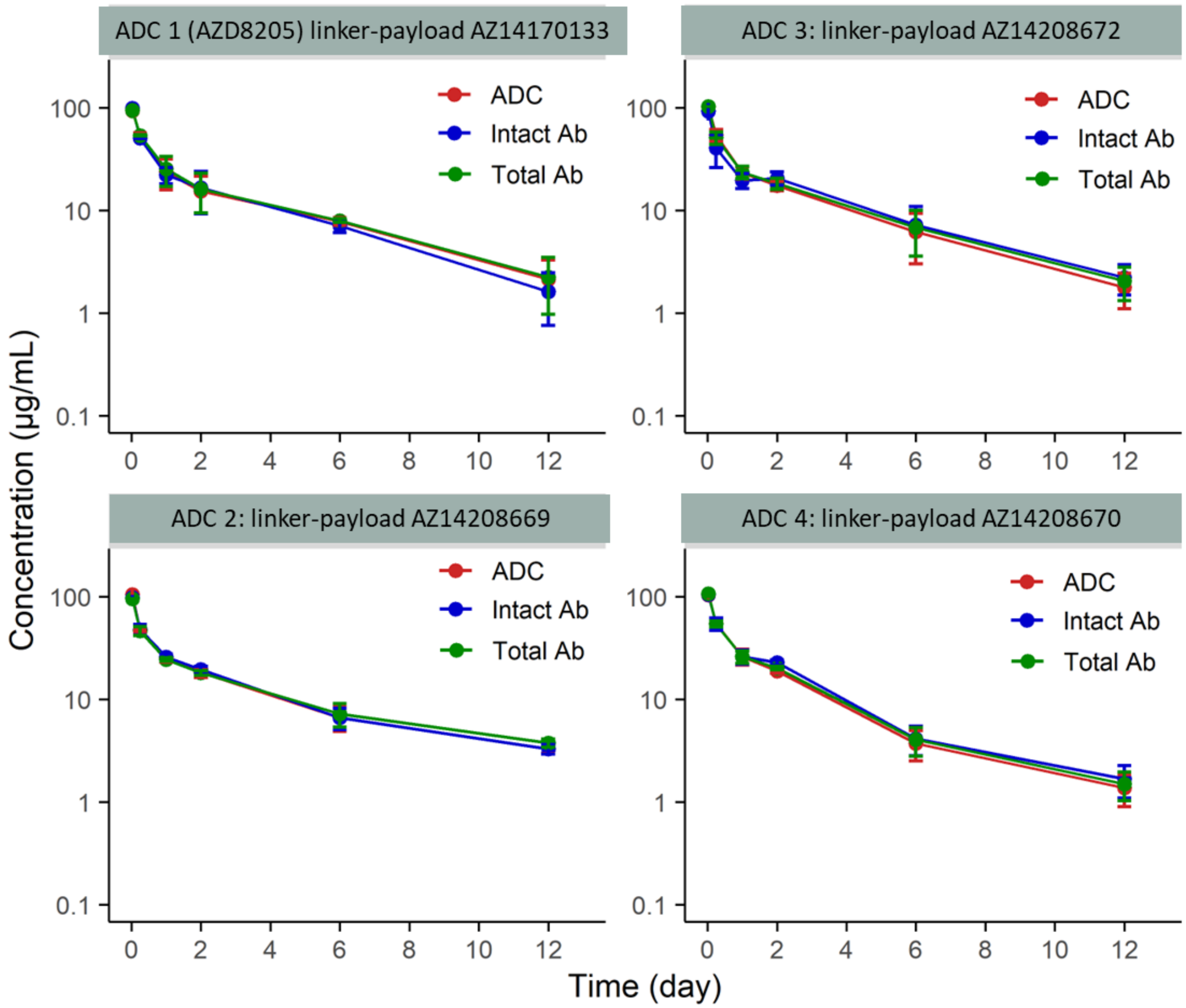

Figure S2. Concentration-time profiles of ADC1-4 after a single bolus 5 mg/kg IV dose in Tg32 mice analyzed by LBA-LC-MRM. The ADC assay captures with the anti-human Fc antibody and detects the PL. The ADC concentration here included both the hydrolyzed and non-hydrolyzed linker

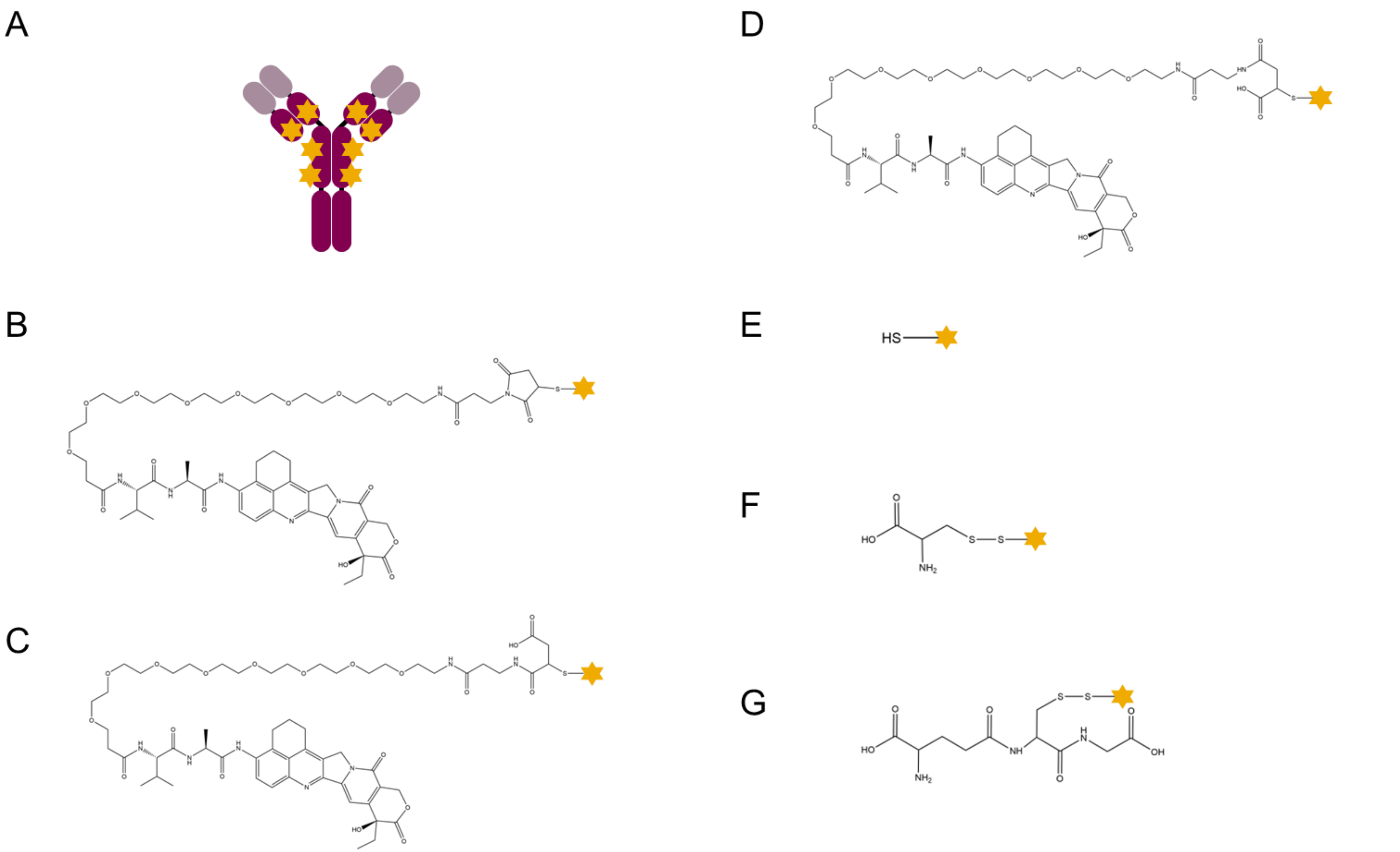

Figure S3. (A) Conjugation sites of linker-PL on anti-B7-H4 antibody marked with yellow stars. Structures of parent and biotransformed species. (B) Thio-succinimide conjugated linker-PL (from ADC 1). (C and D) Isomers of hydrolyzed thio-succinimide conjugated linker-PL (from ADC 1). (E) Liberated thiol (after linker-PL deconjugation) on antibody. (F) Cysteine adduct forming disulfide bond with liberated thiol on antibody. (G) GHS adduct forming disulfide bond with liberated thiol on antibody.

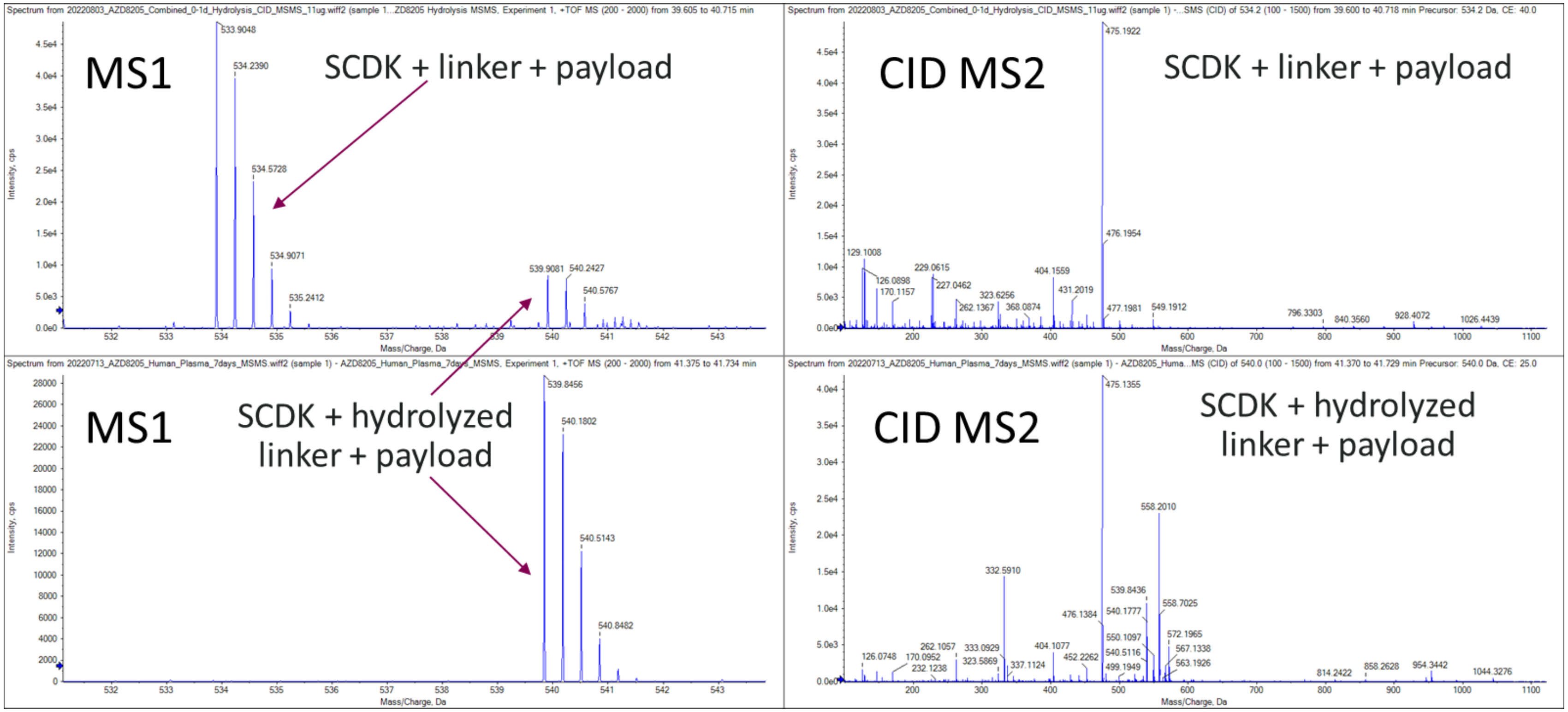

Figure S4. Selected thio-succinimide hydrolysis species confirmed with bottom-up LBA-LC- MRM^{HR}. Peptide SCDK is on the heavy chain peptide with linker and PL, conjugated to the C. The spectra are from in vitro plasma incubation samples with 0day incubation (top panels) and 7 days incubation (bottom panels) at 37 °C. The left panels are the MS1 spectra, where the observed mass matched the theoretical mass in Table S4. The right panels are the CID fragmentation for the non-hydrolyzed and the hydrolyzed. While a signature fragment from the PL section (475) was observed in both spectra, there is a series of +18 fragment ions indicating the hydrolysis of the sample.

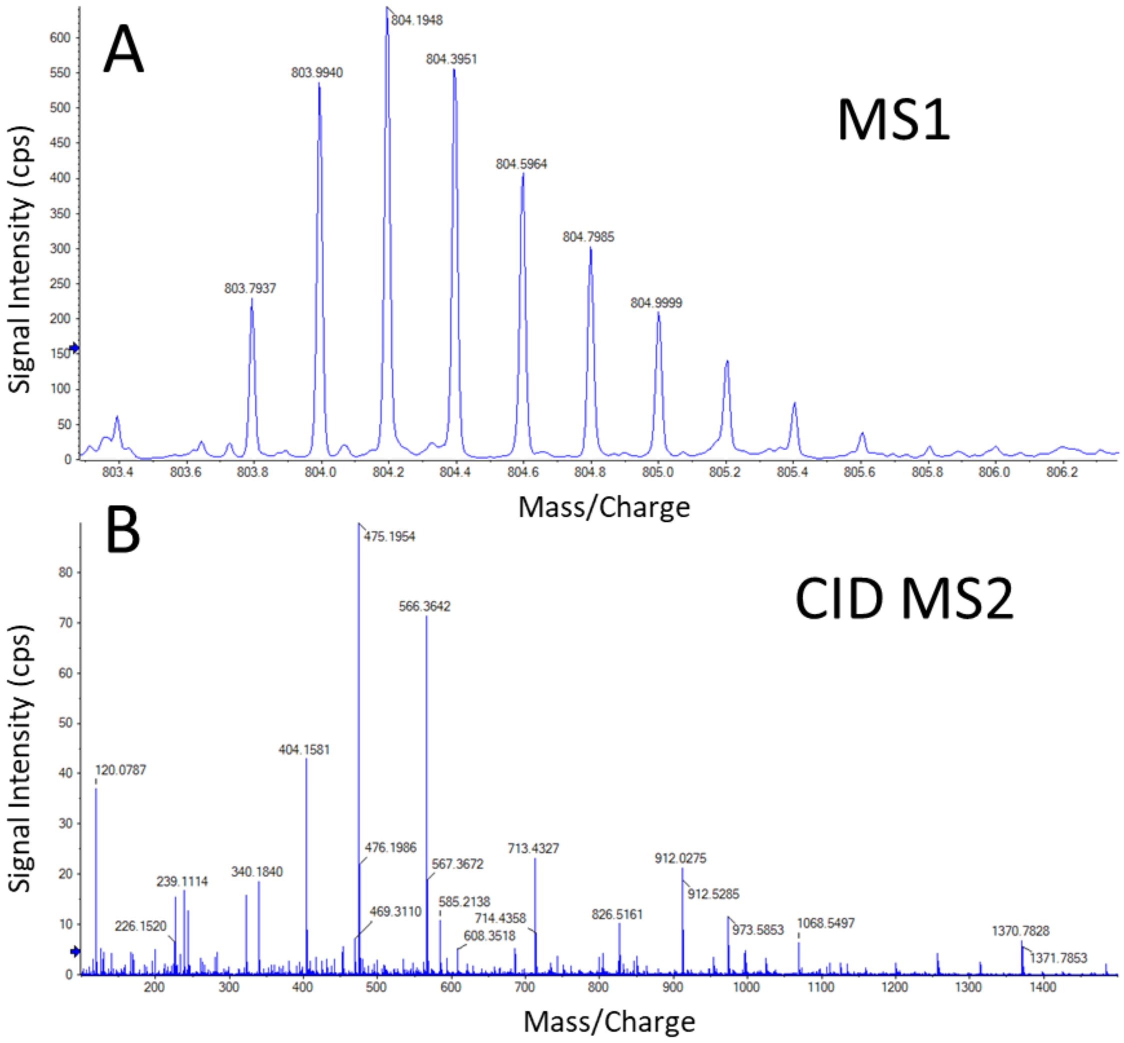

Figure S5. Cysteine adducts confirmed with bottom-up LBA-LC- MRM^{HR}. The spectra are from in vitro plasma incubation samples, 7 days at 37 °C. (A) MS1 spectra for THTCPPCPAPELLGGPSVFLFPPKPK, the hinge region heavy chain peptide, with one cysteine connected to the hydrolyzed linker and PL while the other cysteine connected to a cysteine. (B) MS2 spectra with the signature ion 475 and 404 from the PL, and peptide fragment series for the peptide backbone.

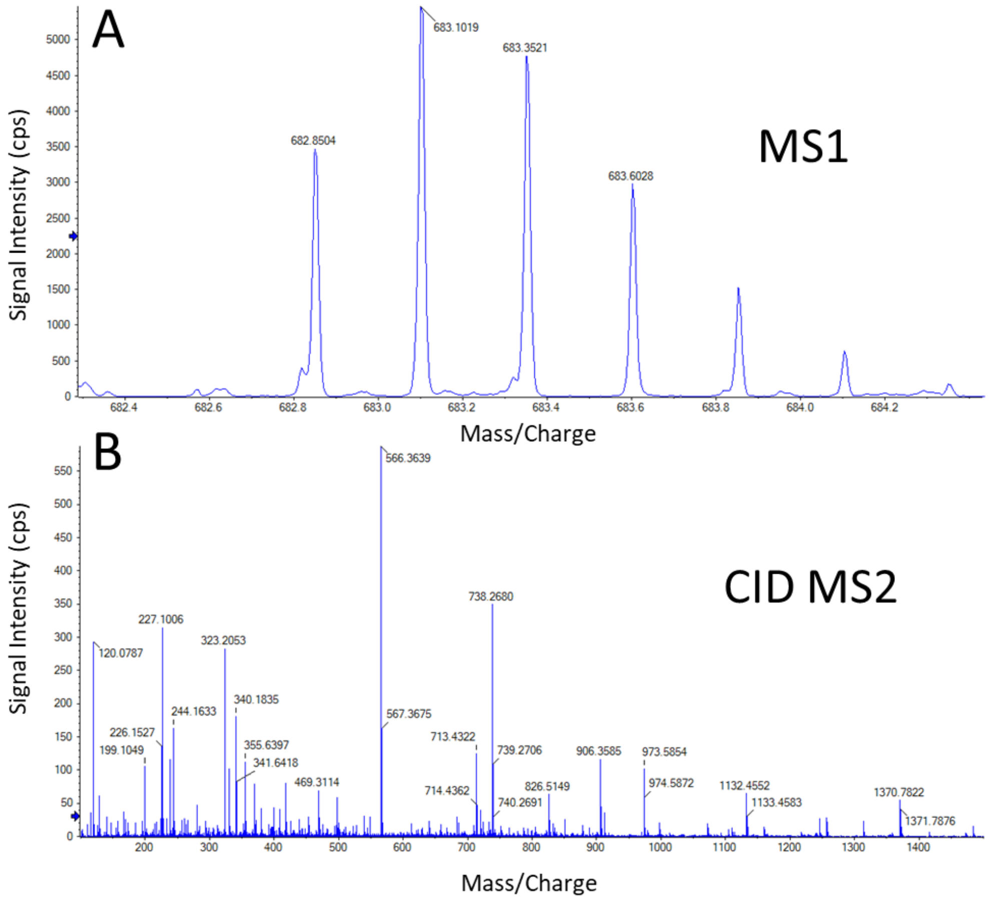

Figure S6. Reformation of intra-chain disulfide bonds confirmed with bottom-up LBA-LC- MRM^{HR}. The spectra are from in vitro plasma incubation samples, 7 days at 37 °C. (A) MS1 spectra for THTCPPCPAPELLGGPSVFLFPPKPK, the hinge region heavy chain peptide, with the two cysteines forming an intra-chain disulfide bond. (B) MS2 spectra with the peptide fragment series for the peptide backbone.

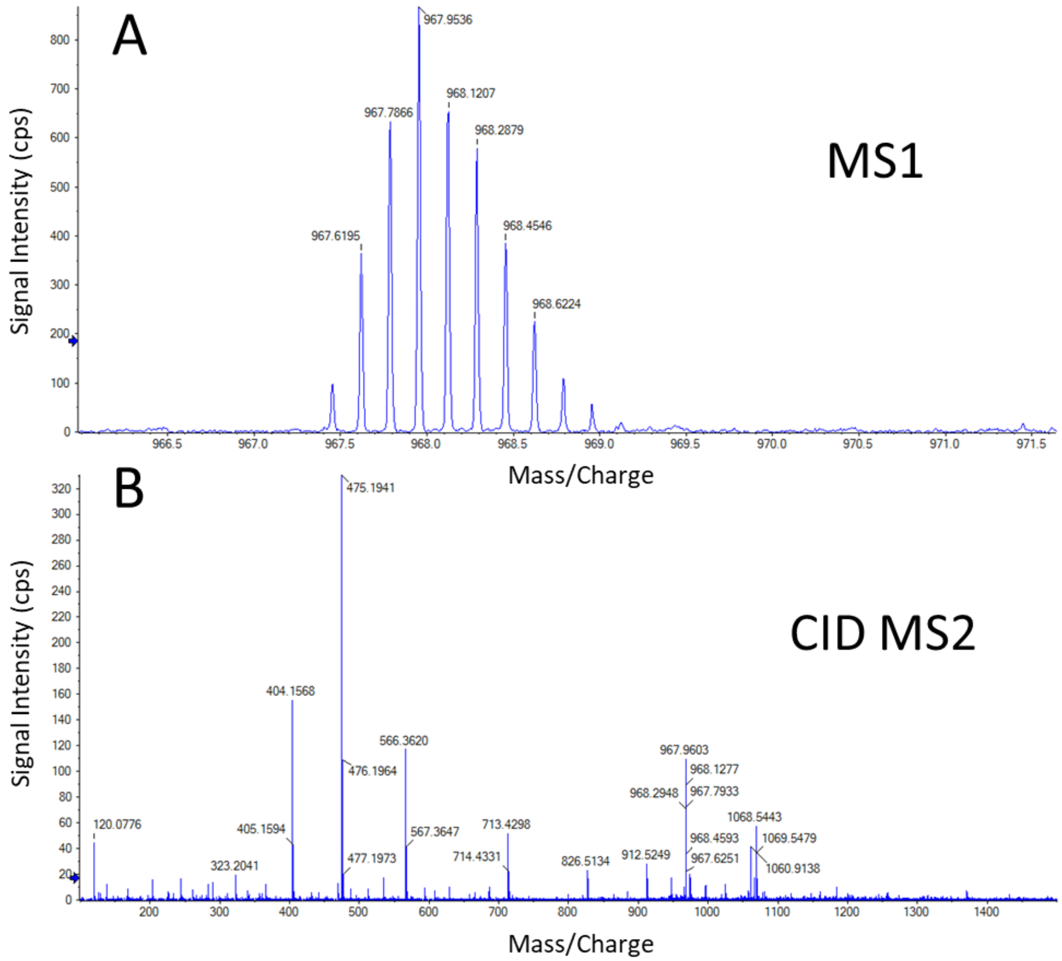

Figure S7. Reformation of inter-chain disulfide bonds confirmed with bottom up LBA-LC- MRM^{HR}. The spectra are from in vitro plasma incubation samples, 7 days at 37 °C. (A) MS1 spectra for GE***C***__S***C***DKTHTCPPCPAPELLGGPSVFLFPPKPK, the hinge region peptides, with the two cysteines from light chain (GE***C***) and heavy chain (S***C***DK) forming an inter-chain disulfide bond, and two other cysteines (CPPC) connected to hydrolyzed linker with PL (B) MS2 spectra with the peptide fragment series for the peptide backbones and the signature ions 475 and 404 from the PL.

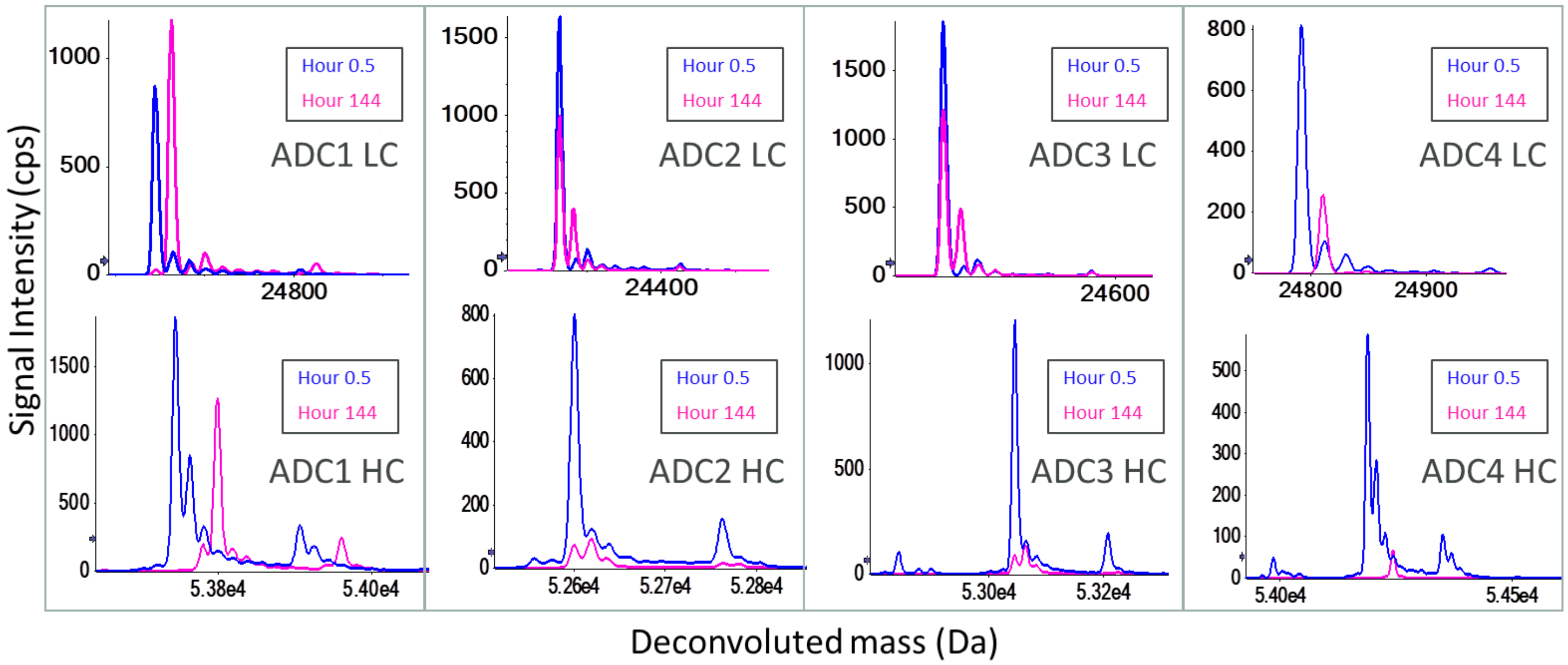

Figure S8. Thio-succinimide hydrolysis at two time points post-dose for the light chain (top) and heavy chain (bottom) for ADCs 1-4, shown with deconvoluted spectra at the time of elution.

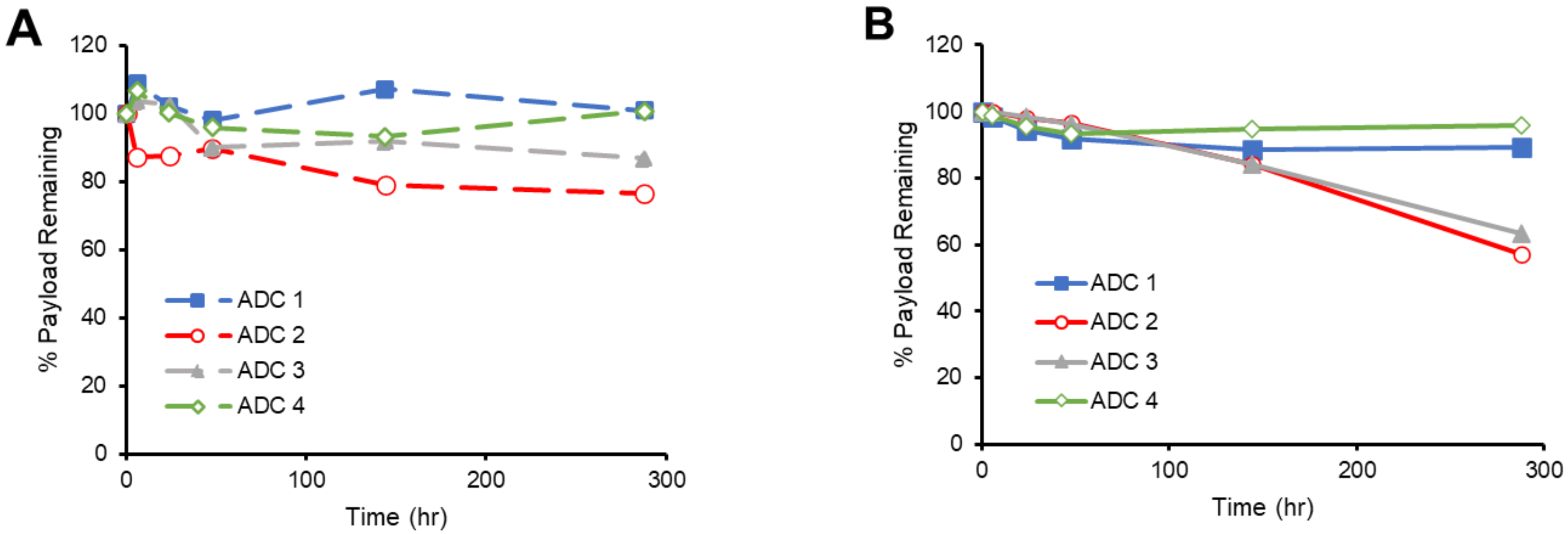

Figure S9. Relative quantification for DAR with LC-MRM method (A) and intact LC-HRMS method (B) for ADC1-4. For intact LC-HRMS method, % DAR for each heavy and light chain species separately was calculated by dividing the sum of the peak areas of all species that have the same DAR by the sum peak area of all species per chain. Then, the % DAR for each species is converted to average DAR for each timepoint, and light and heavy chain DAR values are summed. % Payload Remaining was calculated by normalizing each timepoint to first timepoint (0.5 hr).

Examples of formulae to calculate DAR using LC-HRMS approach are shown below:

$$\%\ chain\ DAR = \frac{Sum\ peak\ areas\ of\ specific\ DAR\ species}{Sum\ peak\ areas\ of all\ species} \times 100\%$$

$$Average\ DAR\ for\ light\ chain = \%\ light\ chain\ DAR\ 1$$

$$Average\ DAR\ for\ heavy\ chain = (\%\ heavy\ chain\ DAR\ 1) + (\%\ heavy\ chain\ DAR\ 2\ \times 2) + (\%\ heavy\ chain\ DAR\ 3\ \times 3)$$

$$Average\ DAR\ of\ ADC = Average\ DAR\ of\ LC + Average\ DAR\ of\ HC$$

Examples of formulae to calculate DAR for -LC-MRM approach are shown below

$$\%\ Payload\ remaining = \frac{Concentration\ of\ ADC}{Concentration\ of\ Total\ Ab} \times 100\%$$

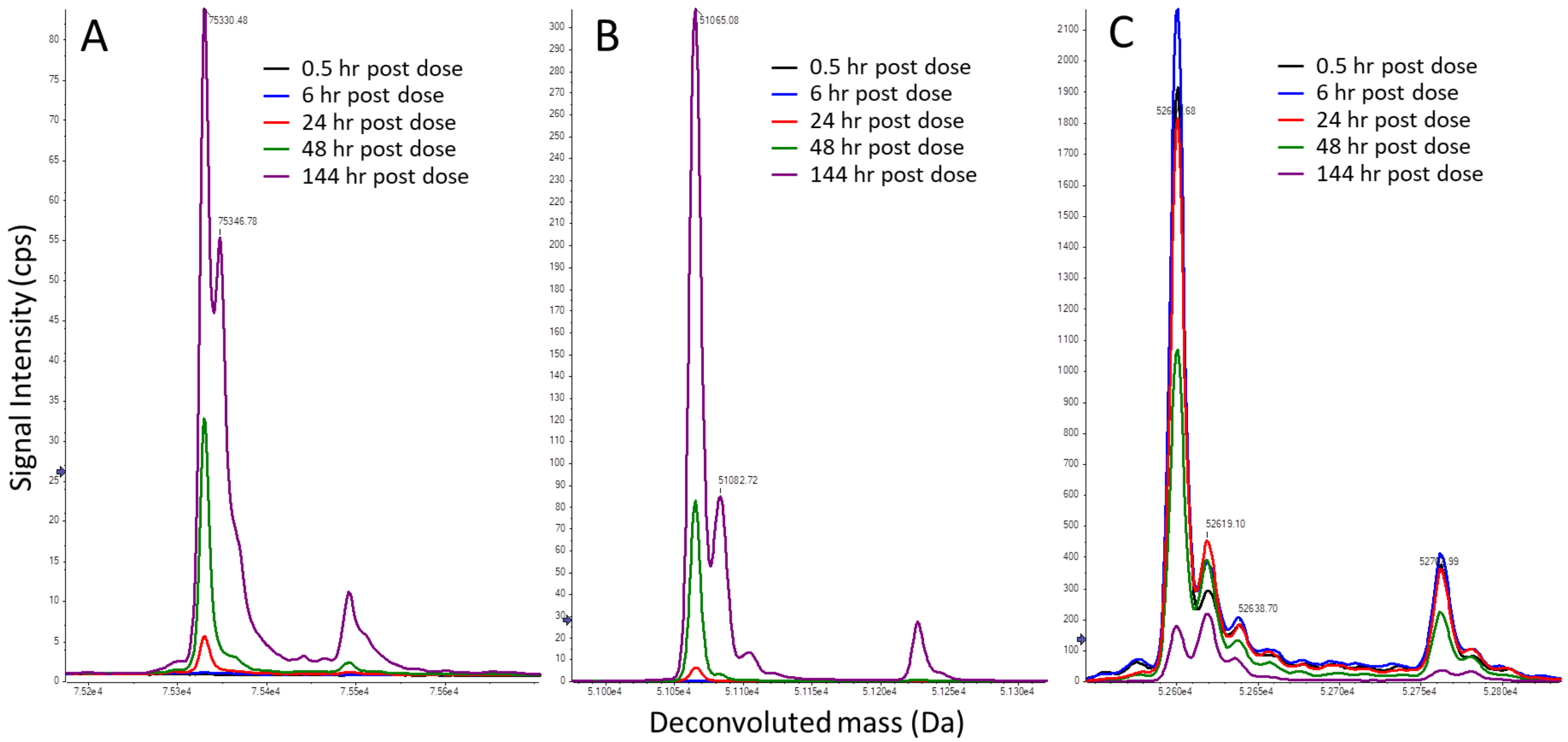

Figure S10. Deconvoluted mass spectra, zoomed in for (A) LC-HC reformation, (B) heavy chain species with only 1 PL and (C) heavy chain species with 2 remaining PLs.

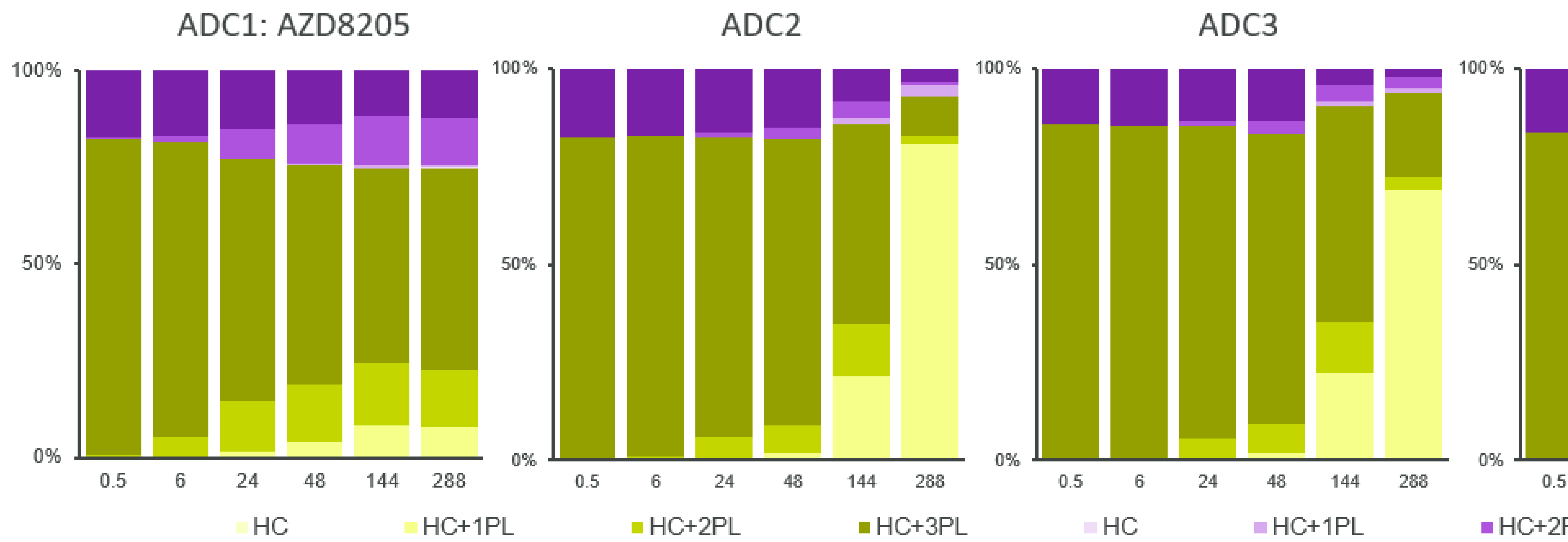

Figure S11. Glycosylation profile of ADCs 1-4. The yellow color is all the G0F species, the purple represented the G1F species. No significant change observed for the glycosylation profiles over the time.
